# Epithelial Salivary Gland Tumors in Ahvaz, Southwest of Iran

**DOI:** 10.5681/joddd.2010.030

**Published:** 2010-12-21

**Authors:** Saede Atarbashi Moghadam, Fazele Atarbashi Moghadam, Mehdi Dadfar

**Affiliations:** ^1^ Assistant Professor, Department of Oral and Maxillofacial Pathology, Faculty of Dentistry, Jundishapur University of Medical Sciences, Ahvaz , Iran; ^2^ Assistant Professor, Department of Periodontics, Faculty of Dentistry, Shahid Sadoughi University of Medical Sciences, Yazd , Iran; ^3^ Private Practice, Tehran, Iran

**Keywords:** Epithelial salivary gland tumors, major salivary gland tumors, minor salivary gland tumors, salivary gland

## Abstract

**Background and aims:**

Salivary gland tumors are rare and specific lesions. There are differences in the incidence and frequency of salivary tumors in both minor and major salivary glands in different countries. This study was carried out to determine the prevalence of major and minor salivary gland tumors in Ahvaz in the south of Iran.

**Materials and methods:**

All the cases, recorded in Imam Khomeyni Hospital and Shafa Hospital, Ahvaz, Iran from 1997 to 2008 were assessed. Age, gender, anatomical location, and histology of all the specimens were evaluated.

**Results:**

Of 112 cases, 84 cases were benign and 28 cases were malignant. Female predominance was observed in these cases. Most lesions appeared in the third to fifth decades of life (60.71%). The incidence of malignant salivary gland tumors increased with age and male predominance was found in malignant tumors. The majority of the tumors occurred in parotid gland. Pleomorphic adenoma was the most common histological type (65.17%). Mucoepidermoid carcinoma and adenoid cystic carcinoma were the most common malignant tumors.

**Conclusion:**

It was shown that the peak incidence age of all salivary gland tumors was the third to fifth decades, and malignant tumors mostly occurred in the sixth to eighth decades. Female predominance for all the tumors and slight male predominance in malignant tumors were observed. Pleomorphic adenoma was the most common benign tumor. The most common malignant tumors were mucoepidermoid carcinoma and adenoid cystic carcinoma.

## Introduction

Salivary gland neoplasms are rare lesions and represent less than 1% of all tumors and 3-6.5% of all head and neck tumors.^1,2^ The parotid and minor salivary glands account for the majority of these tumors.^2^ Despite the relatively low frequency of these tumors, they represent a heterogeneous group of neoplasms, with a broad range of histological types and growth patterns.^1,3^

Reports from different countries of the world have shown differences in the incidence and frequency of salivary tumors in both minor and major salivary glands. Epidemiologic studies of frequency of salivary gland tumors in different countries have reported a range from 0.4 to 13.5 cases per 100,000 inhabitants annually.^3-7^ Studies in this regard are sparse, especially in Asia.^4^

The purpose of this study was to determine the demographic features, tumor location, and histopathological types of patients with salivary gland tumors in Ahvaz in the-south of Iran.

## Materials and Methods

Files of the Department of Pathology, Imam Khomeyni Hospital and Shafa Hospital, Ahvaz, Iran from 1997 to 2008 were retrieved and the cases histologically recorded as salivary gland tumors of epithelial origin were enrolled in this study.

The histology of all the tumors was reviewed and classified according to the World Health Organization (WHO) Histological Typing of Salivary Gland Tumors.^8^ Information regarding age, gender, and anatomical location were determined for each salivary tumor type. The frequencies of different benign and malignant salivary tumors in both major and minor glands were identified. Based on these data, a descriptive statistical analysis was performed using SPSS software.

## Results

The total number of salivary gland tumor cases from 1997 to 2008 was 112 cases. The benign-to-malignant ratio in all the cases was 3:1. The benign-to-malignant ratio in major and minor salivary gland tumors were 3.75:1 and 1.28:1, respectively. There were 62 females (49 benign and 13 malignant) and 50 males (35 benign and 15 malignant) (F:M = 1.24: 1). The peak incidence for all salivary gland tumors extended from the third to eighth decades of life. The most benign tumors occurred among 40-49 year-old patients (21 cases), followed by 30-39 year-olds (20 cases), and 20-29 year-olds (17 cases). It was observed that most malignant tumors occurred among 70-79 year-old patients (8 cases). Six cases (5.35%) were found in 10-19 year-old group and all were benign. Table 1 represents the frequency of benign and malignant tumors, considering the age range.

**Table 1:** Frequency (percent) of benign and malignant tumors, considering the age ranges

Age range (years)	Benign	Malignant	Total
10-19	6 (5.35)	-	6 (5.35)
20-29	17 (15.17)	6 (5.35)	23 (20.53)
30-39	20 (17.85)	3 (2.67)	23 (20.53)
40-49	21 (18.75)	1 (0.89)	22 (19.64)
50-59	7 (6.25)	5 (4.46)	12 (10.71)
60-69	6 (5.35)	3 (2.67)	9 (8.03)
70-79	7 (6.25)	8 (7.14)	15 (13.39)
80-89	—	1 (0.89)	1 (0.89)
90-99	—	1 (0.89)	1 (0.89)

The majority of the tumors occurred in the parotid gland with 83 cases, followed by minor salivary glands, submandibular gland, and sublingual gland (Table 2). Palate was the most frequent site for minor salivary gland tumors (75%). Pleomorphic adenoma was the most common histological type with 73 cases (65.17%) and Warthin’s tumor was the second most frequent with 10 cases (8.92%). Mucoepidermoid carcinoma and adenoid cystic carcinoma were the most common malignant tumors with 9 (8.03%) and 8 (7.14%) cases, respectively. In this study mucoepidermoid carcinoma and adenoid cystic carcinoma accounted for 32.14% and 28.57% of all malignant tumors. Table 3 represents the frequency of salivary gland tumors based on pathologic diagnosis.

**Table 2:** Frequency of benign and malignant tumors, considering the types of involved salivary gland

Tumor location	Benign N (%)	Malignant N (%)	Total Number	Percent (%)
Parotid gland	66 (78.57)	17 (60.71)	83	74.10
Minor salivary glands				
Palate	7 (8.33)	5 (17.85)	12	10.71
Buccal	2 (2.38)	2 (7.14)	4	3.57
Submandibular gland	9 (10.71)	3 (10.71)	12	10.71
Sublingual gland	—-	1 (3.57)	1	0.89
Total	84 (100)	28 (100)	112	100

**Table 3:** Frequency of salivary gland tumors on the basis of pathologic diagnosis and site of tumors

Pathologic diagnosis	Parotid	Submandibular	Sublingual	Palate	Buccal	Total Number	Percent (%)
Pleomorphic adenomas	55	9	—	7	2	73	65.17
Warthin’s tumor	10	—	—	—	—	10	8.92
Mucoepidermoid carcinoma	7	1	—	1	—	9	8.03
Adenoid cystic carcinoma	4	1	—	3	—	8	7.14
Adenocarcinoma, NOS	2	—	1	—	—	3	2.67
Polymorphous low-grade adenocarcinoma	—	—	—	1	2	3	2.67
Salivary duct carcinoma	2	—	—	—	—	2	1.78
Oxyphilic adenoma	1	—	—	—	—	1	0.89
Squamous cell carcinoma	2	1	—	—	—	3	2.67

## Discussion

In this study, the benign-to-malignant ratio was 3:1. Most studies have shown a predominance of benign over malignant tumors with the ratio ranging from 1.18:1 to 5.62:1 in different populations.^3,5,9-11^ However, a study by Tilakaratne et al^4^ showed an equal frequency of benign and malignant salivary gland tumors in Sri Lanka. Higher incidence of benign tumors compared to malignant tumors was also observed in minor salivary glands (Table 1) consistent with the results of studies carried out by Buchner et al and Toida et al.^12,13^

The female-to-male ratio of all the cases was 1.24:1. Female predominance was observed in benign tumors (1.4:1), but there was a slight male predominance in malignant tumors (F:M = 1:1.15). Tilakarrante et al^4^ showed female predominance in both benign and malignant tumors while Li et al^3^ showed male predominance in all the tumors and female predominance in benign tumors. Licitra et al^14^ showed equal sex distribution of malignant tumors, but male predominance (1.3:1) for all the salivary gland tumors.

Most lesions appeared in the third to fifth decades of life (60.71%). The peak incidence age of benign tumors was forth and fifth decades (36.60%); that of malignant tumors was sixth to eighth decades (14.28%). Earlier studies have shown a similar age range for benign tumors but the average range for malignant tumors in those studies have been reported to be the fourth and fifth decades.^3,4^ In this study 5.35% of tumors occurred under 20 years of age and all of them were benign tumors (Table 1). Neoplastic changes are very rare in children and adolescents and not more than 5% of all salivary gland tumors are found in the >16 age group.^15^

The majority of the tumors occurred in parotid gland (74.10%), followed by the minor salivary glands of the palate and submandibular gland with the same frequency (10.71%) (Table 2). These findings were consistent with other studies.,^9-11,16^ Only one case of sublingual involvement was found, which was malignant. On the whole, 14.28% of all the tumors involved minor salivary glands. Considering major series, epithelial tumors of minor salivary glands accounted for 9-23% of all salivary gland tumors.^12^ In the present study 75% of all minor salivary gland tumors occurred in the palatal mucosa. This finding was similar to earlier studies, which have reported the palate as the most common site for minor salivary tumors, in which 42-75% of tumors occurred.^12,17,18^ The second most common site of minor salivary gland tumors in this study was buccal mucosa. A study carried out by Li et al^3^ also showed the buccal region as the second location for minor salivary gland tumors followed by the floor of the mouth and lips.

Pleomorphic adenoma was the most common histological type (65.17%) (Table 3). Pleomorphic adenoma has been listed universally as the most common tumor in all the studies with a range of 40-72%.^3-7^ Warthin’s tumor was the second most frequent tumor (8.03%) and all of them were found in the parotid gland (Table 3). Some studies have reported Warthin’s tumor as the second most common benign tumor but in all the salivary gland tumors they have found mucoepidermoid carcinoma as the second most common tumor.^3,4^ According to literature review, Warthin’s tumor accounts for 4-13% of all salivary tumors and is mainly found in major salivary glands, particularly the parotid gland.^19^

Mucoepidermoid carcinoma and adenoid cystic carcinoma were the most common histological type of malignant tumors (8.03% and 7.14% of all the tumors and 32.14% and 28.57% of all the malignant tumors, respectively) (Table 3). Other studies have reported mucoepidermoid carcinoma as the most frequent malignant tumors.^3,4,12,18^ Some studies have reported that adenoid cystic carcinoma is more common than mucoepidermoid carcinoma.,,^20,21^ It might be attributed to the fact that before 1991 some polymorphous low-grade adenocarcinomas might have been included in the group of adenoid cystic carcinoma, which increased its frequency.^3,4^ Regarding the fact that some studies after 1991 also reported adenoid cystic carcinoma as the most common malignant tumor and distinguished it from polymorphous low-grade adenocarcinoma,^7,13^ the predominance of adenoid cystic carcinoma might be attributed to geographic and ethnic differences.^12^

In this study mucoepidermoid carcinoma was the most common malignant tumor of major salivary glands. Adenoid cystic carcinoma and polymorphous low-grade adenocarcinoma were the most common malignant tumors of minor salivary glands (Table 3). Toida et al^13^ and Wang et al^22^ found higher numbers of adenoid cystic carcinoma in minor salivary glands. However, some studies have reported higher prevalence rates for mucoepidermoid carcinoma in minor salivary gland tumors.,^12^ In this study, polymorphous adenocarcinoma was the third most common malignant tumor and was only found in minor salivary glands. Other studies have confirmed polymorphous adenocarcinoma as the third most common malignant salivary gland tumor.^4^

In conclusion, this study collected the epidemiologic data about age, gender, location, and histopathological classification of major and minor salivary gland tumors in the southwest of Iran. It was shown that the peak incidence ages of all salivary gland tumors were the third to fifth decades, and malignant tumors mostly occurred in the sixth to eighth decades. Female predominance for all the tumors and slight male predominance in malignant tumors were observed. We hope these data in combination with data from other parts of Iran could assist in the early diagnosis and, as a result, in the early treatment of salivary gland tumors.

## References

[1] Pons-Vicente O, Almendros-Marqués N, Berini-Aytés L, Gay Escoda C. Minor salivary gland tumors:A clonicopathological study of 18 cases. Med Oral Pathol Oral Cir Bucal 2008;13:E582-8. 18758404

[2] Ethunandan M, Davies B, Pratt CA, Puxeddu R, Brennan PA (2009). Primary epithelial submandibular salivary gland tumors—Review of management in a district general hospital setting. Oral Oncol.

[3] Li LJ, Li Y, Wen YM, Liu H, Zhao HW (2008). Clinical analysis of salivary gland tumor cases in west china in past 50 years. Oral Oncol.

[4] Tilakaratne WM, Jayasooriya PR, Tennakoon TM, Saku T (2009). Epithelial salivary tumors in Sri Lanka: a retrospective study of 713 cases. Oral Surg Oral Med Oral Pathol Oral Radiol Endod.

[5] Przewozny T, Stankiewicz C (2004). Neoplasms of the parotid gland in northern Poland, 1991-2000: an epidemiologic study. Eur Arch Otorhinolaryngol.

[6] Speight PM, Barrett AW (2002). Salivary gland tumors. Oral Dis.

[7] Ito FA, Ito K, Vargas PA, de Almeida OP, Lopes MA (2005). Salivary gland tumors in Brazilian population: a retrospective study of 496 cases. Int J Oral Maxillofac Surg.

[8] Barnes L, Eveson JW, Reichart P, Sidransky D. World Health Organization classification of tumors. Pathology and genetics, head and neck tumors. Lyon: IARC Press; 2005:209-81.

[9] Otoh EC, Johnson NW, Olasoji H, Danfillo IS, Adeleke OA (2005). Salivary gland neoplasms in Maiduguri, north-eastern Nigeria. Oral Dis.

[10] Kamulegeya A,  Kasangaki  Kasangaki (2004). Neoplasms of the salivary glands: a descriptive retrospective study of 142 cases-Mulago hospital Uganda. J Contemp Dent Pract.

[11] Ma'aita JK, Al-Kaisi N, Al-Tamimi S, Wraikat A (1999). Salivary gland tumors in Jordan: a retrospective study of 221 patients. Croat Med J.

[12] Buchner A, Merrell PW, Carpenter WM (2007). Relative frequency of intra-oral minor salivary gland tumors: a study of 380 cases from northern California and comparison to reports from other parts of the world. J Oral Pathol Med.

[13] Toida M, Shimokawa K, Makita H, Kato K, Kobayashi A, Kusunoki Y (2005). Intraoral minor salivary gland tumors:a clinicpathological study of 82 cases. Int J Oral Maxillofac Surg.

[14] Licitra L, Grandi C, Prott FJ, Schornagel JH, Bruzzi P, Molinari R (2003). Major and minor salivary gland tumors. Cirt Rev Oncol Hematol.

[15] Ellies M, Schaffranietz F, Arglebe C, Laskawi R (2006). Tumors of the salivary glands in childhood and adolescence. J Oral Maxillofac Surg.

[16] Lima SS, Soares AF, Amorim RF, Freitas Rde A. (2005). Epidemiologic profile of salivary gland neoplasms: analysis of 245 cases.. Rev Bras Otorrinolaringol (Eng Ed).

[17] Mücke T, Robitzky LK, Kesting MR, Wagenpfeil S, Holhweg-Majert B, Wolff KD, et al (2009). Advanced malignant minor salivary gland tumors of the oral cavity. Oral Surg Oral Med Oral Pathol Oral Radiol Endod.

[18] Dhanuthai K, Boonadulyarat M, Jaengjongdee T, Jiruedee K (2009). A clinico-pathologic study of 311 intra-oral salivary gland tumors in Thais. J Oral Pathol Med.

[19] Maiorano E, Lo Muzio L, Favia G, Piattelli A (2002). Warthin’s tumor:a study of 78 cases with emphasis on bilaterality, multifocality and association with other malignancies. Oral Oncol.

[20] Everson JW, Cawson RA (1985). Tumors of minor (oropharyngeal) salivary glands: a demographic study of 336 cases. J Oral Pathol.

[21] Isacsson G, Shear M (1983). Intraoral Salivary gland tumors: a retrospective study of 201 cases. J Oral Pathol.

[22] Wang D, Li Y, He H, Liu L, Wu L, He Z (2007). Intraoral minor salivary gland tumors in Chinese population: a retrospective study of 737 cases. Oral Surg Oral Med Oral Pathol Oral Radiol Endod.

